# Economic burden attributable to high BMI-caused cancers: a global level analysis between 2002 and 2021

**DOI:** 10.1186/s12916-025-04109-8

**Published:** 2025-05-28

**Authors:** Jiacheng Zheng, Laiang Yao, Katie Lei, Wanying Huang, Yi Jie Zhou Luo, Priscilla Hui–Xuan Tran, Aaron Guan, Yueyi Qiu, Yusuff Adebayo Adebisi, Don Lucero-Prisno Eliseo, Claire Chenwen Zhong, Martin C.S. Wong, Junjie Huang, Global Health Focus Epidemiology Group (GHFEG)

**Affiliations:** 1 Birmingham Business School, College of Social Science, University of Birmingham, Birmingham, UK; 2 The Jockey Club School of Public Health and Primary Care, Faculty of Medicine, The Chinese University of Hong Kong, Hong Kong SAR, China; 3 Centre for Health Education and Health Promotion, Faculty of Medicine, The Chinese University of Hong Kong, Hong Kong SAR, China; 4 School of Philosophy, Psychology and Language Sciences, University of Edinburgh, Edinburgh, Scotland, UK; 5 Department of Mathematics and Statistics, McGill University, Montreal, QC Canada; 6 Schulich School of Medicine and Dentistry, University of Western Ontario, London, ON Canada; 7 Department of Biology, Faculty of Science, University of Western Ontario, London, ON Canada; 8 Department of Pharmacology and Toxicology, University of Toronto, Toronto, ON Canada; 9 Department of Psychology, Faculty of Science, University of Alberta, Edmonton, AB Canada; 10 Nuffield Department of Population Health, University of Oxford, Oxford, UK; 11 Department of Global Health and Development, London School of Hygiene and Tropical Medicine, London, UK

**Keywords:** High BMI-caused cancers, Economic burden, Value of Statistical Life Approach, Different income tiers

## Abstract

**Background:**

Obesity and overweight are prevailing concerns in modern society, but high BMI shows an established correlation with the risk of cancers that impacts not only medical issues but also economic performance. This study analyzes the economic loss due to high BMI-caused cancers (HBCCs).

**Methods:**

This study used the comprehensive Global Burden of Disease (GBD) 2021 database and estimated the economic loss of HBCCs through the Value of Statistical Life approach (VSLA), incorporating a willingness-to-pay metric. Health burdens are expressed in age-standardized DALYs and death rates, and economic burdens are shown in dollars lost (2021 PPP) calculated from total DALYs. A joinpoint regression analysis was utilized to capture the temporal trends, cancer incidence, and economic losses attributed to high BMI across various countries and income levels. We calculated the average annual percentage change (AAPC) in total economic loss to evaluate the trend over the study period.

**Results:**

There is a growing trend in both economic loss and disease burden of HBCCs on a global level. Colon and rectum cancer (CRC) show the highest economic loss ($2593.159 million, UI: 1109.04–4119.61, to $7294.52 million, UI: 3134.75–11,511.13), with pancreatic (AAPC: 10.47*, CI: 8.01–13.51) and liver cancer (AAPC: 8.08*, CI: 5.77–10.35) being the fastest growing cause. The cancer burden for all measures positively correlates with the country’s income level; high-income countries are the only group to experience a decreasing trend in the health burden, but they are still increasing in economic burden. Differences in loss of certain types of cancer and gender gap are observed in different income tiers.

**Conclusions:**

These findings indicate a significant upward trend in economic loss, highlighting the urgency for strengthened policy measures. It is crucial for policymakers to implement effective risk reduction and resilience-building strategies to mitigate future economic loss and better protect vulnerable communities.

**Supplementary Information:**

The online version contains supplementary material available at 10.1186/s12916-025-04109-8.

## Background

Numerous epidemiological studies provide consistent evidence that a high body mass index strongly correlates with certain cancers’ incidence, exacerbates the established cancer, and erodes the efficacy of the therapy [1]. The link between obesity and cancer incidence, prevalence, and mortality has also been discussed through mechanism analysis [2]. Notwithstanding, factors like the sedentary lifestyle, erratic hours, and greasy diet, which are common in the modern world, construct a hotbed for obesity to prevail. Previous studies indicate that approximately 671 million individuals suffer from excess body weight, and the prevalence is still estimated to increase [3, 4]. These facts portend that the cancer burden attributable to overweight would become increasingly severe in the following decades.

Meanwhile, it is crucial to understand that cancers influence not only patients but also families, employers, and the social welfare system, necessitating a comprehensive approach to measure its burden from medical, epidemiological, and socioeconomic perspectives. Labor supply loss is a case in point. Mortality and morbidity lead to the reduction in human capital by reducing the population, increasing absenteeism, and the fact that households with affected family members divert a significant part of their time to give care [5]. Nevertheless, current studies doing global-level analysis of HBCCs, like Fitzmaurice [6], only describe the death trend and DALYs, rarely investigate economic loss, and their datasets were not the most updated. Nevertheless, current global analyses of HBCCs (e.g., Fitzmaurice et al.) [6] have mostly described trends in deaths and DALYs, rarely examining the economic loss, and often using outdated data. Moreover, traditional human capital methods only account for direct productivity losses, whereas a willingness-to-pay-based approach can capture the broader economic value of life lost.

This study aims to fill the gap by evaluating the disease burden of HBCCs worldwide from epidemiologic and economic perspectives, considering genders and the differences between regions due to diverse geographical backgrounds. Unlike productivity loss approaches that focus solely on forgone wages, the Value of Statistical Life Approach (VSLA) incorporates individuals’ willingness to pay to reduce mortality risk, thereby capturing the intrinsic value of life beyond labor market contributions. It will assess the age-standardized disability-adjusted life years (DALYs) rate, death rate, financial economic loss, and temporal trend.

## Methods

### Study design

We first utilized the GBD 2021 database (https://vizhub.healthdata.org/gbd-results/) to extract DALYs, morbidity, and mortality associated with HBCCs across 185 countries from 2002 to 2021 [7]. High BMI-caused cancers were defined using GBD’s comparative risk assessment framework; we extracted, for each relevant cancer, the portion of DALYs and deaths attributable to high BMI. Not all cases of these cancers are considered high BMI-attributable. Instead, we used GBD’s estimated proportion of each cancer type linked to high BMI based on established risk assessments. This method ensures that our analysis captures only the obesity-attributable fraction of cancer burden, rather than assuming all cases are caused by obesity. We did macroscopic descriptive statistics in an epidemiologic sense based on the difference between sexes, the diversity in country groups with different income levels (categorized by the World Bank) [8], and the temporal trends from 2002 to 2021.

To simplify the problem, this study focuses on the economic burden of high BMI-caused cancers in a broader societal context, beyond labor supply and lost productivity. While economic loss due to cancer-related mortality and morbidity is traditionally evaluated using the human capital approach, this method does not fully capture the intrinsic value of life and health. Instead, we employed the Value of a Statistical Life Approach (VSLA), which extends beyond labor market valuation by incorporating individuals’ willingness to pay for mortality risk reduction. The details of the transformation from DALYs to monetary loss are shown in the statistical analysis part. Subsequently, joinpoint regression was then applied to capture the temporal trends, and comparisons between 2000, 2002, and 2021 were made over the diversity in changes in different income groups, data in different sexes, different types of cancers, and the difference in health and economic burden. Health burdens are expressed in age-standardized DALYs and death rates, and economic burdens are shown in dollars lost (2021 PPP) calculated from total DALYs.

### Data sources

This study was conducted using data from GBD 2021, which aims to estimate the burden caused by 369 diseases and 87 risk factors in 204 countries/territories and help establish disease control strategies globally. Previous studies have described the detailed methodologies of GBD 2021 and the comparative risk assessment specifically for high BMI [9]. Briefly, data were collected from population-based cancer registries, vital registration systems, or verbal autopsy studies. The GBD 2021 studies defined cancers based on the WHO standard. GBD’s comparative risk assessment methodology quantifies the proportion of each cancer attributable to high BMI by analyzing exposure distributions, relative risks, and counterfactual risk scenarios. This approach ensures that our economic burden estimates account only for obesity-attributable disease burdens. A Cause of Death Ensemble model (CODEm), a form of Bayesian geospatial regression analysis, was utilized to estimate mortality by age, sex, location, and year. DALYs were calculated as the sum of years of life lost and years lived with disability. The percentage of DALYs caused by high BMI for a specific type of cancer was also provided by GBD 2021.

Additional data needed to estimate the economic burden are Gross National Income (GNI) per capita given 2021 purchasing power parity, real exchange rate, life expectancy (LE), and mid-age point (MA), which can be collected from the World Bank [10] and the Department of Economic and Social Affairs of the United Nations [11]. Since only 174 countries or territories had all indicators available during the 20-year period, we supplemented the data with a regression equation for missing indicators versus DALYs if there are only one to two missing indicators. However, for countries or regions where multiple indicators are unavailable, we directly mark them as data loss to ensure the reliability of the data. We finally have data on economic losses for 185 countries and territories. By adjusting for these macroeconomic variables, our estimates reflect real economic conditions across different countries and time periods.

### Statistical analysis

Cancer burden was quantified using disability-adjusted life years (DALYs), consistent with Global Burden of Disease (GBD) methodology. For each cancer case, we calculated years of life lost (YLL) based on age-specific life expectancy and years lived with disability (YLD) by multiplying the illness duration by the appropriate disability weight from the GBD 2019 study. (The disability weights in the newer GBD 2021 are highly comparable, with only minor updates in a few health states; thus, using GBD 2019 weights do not materially affect our results.) All estimates are reported with 95% uncertainty intervals (UI), which were derived from the empirical distribution of the estimates (e.g., via 1000 bootstrap replications)**.** This non-parametric approach defines the 95% UI as the 2.5th to 97.5th percentile range, rather than assuming a normal distribution, thereby more accurately reflecting uncertainty in our model outputs [9]. Similarly, we applied the VSLY calculation to each of the 1000 DALY draws to propagate uncertainty into the economic loss estimates, obtaining 95% UIs for the economic loss as well.

The VSLA reconstructs a person’s implicit valuation of his or her life by estimating a person’s willingness to accept premia for risky occupations via wage regressions or by estimating a person’s willingness to pay for reducing risks via hedonic price regressions [12]. However, traditional VSLA may be inaccurate for policies that mitigate large pandemic risks, such as COVID-19. Following the path of Sweis [13], the VSL is revisited using a different approach and the modified theory of the demand for health by Gary Becker [14]. We utilized the index—Value of Statistical Life per Year (VSLY) multiplied by the total DALYs to indicate the loss from both mortality and morbidity, and we made comparisons.

The functions to calculate VSL and VSLY are shown below and derived by Sweis [13].

1 $$VSL=\frac{1}{r} \cdot \frac{1}{(1+r)}\cdot\frac{1}{\lambda}\cdot({\mathrm x}_1+{\mathrm l}_1{\mathrm w}_1)$$

2 $$VSLY=\frac{VSL}{LE-MA}$$

where *r* is the discount rate, *λ* is the degree of concavity in the single–period utility function, *x*_*1*_ is the total spending on goods X, L_1_ is the leisure time, and *w*_*1*_ is the wage rate. We used the same assumptions by Becker [14] that the total time available for work and leisure is approximately 5200 h per year, excluding the time for sleep, which is around 68 h per week. In this sense, the annual work and leisure hours per year are 1900 and 3300 h, respectively. The value for λ we take is 0.5, as suggested by Sweis [15]. approximately 5200 h per year (after excluding ~ 68 h/week for sleep), with 1900 h devoted to work and 3,300 to leisure. We set λ = 0.5 (as suggested by Sweis [15]) and the discount rate *r* to be each country’s annual *real* interest rate (inflation-adjusted). Using real interest rates as the discount factor ensures that future values are expressed in constant terms, aligning with standard economic evaluation practice. This approach is preferable to using a fixed discount rate, as it captures each country’s prevailing economic conditions (e.g., inflation and growth) rather than applying a uniform assumption across all contexts. *Excel* and *Pandas* conducted the calculation process. In a sensitivity analysis, we also tested fixed discount rates of 3% and 7%. As expected, using a higher rate (7%) produced lower VSL (and economic loss) estimates and a lower rate (3%) produced higher estimates; however, the overall trends and cross-country patterns were not materially affected. For ethical consideration, while the Value of Statistical Life (VSL) approach is widely used in economic evaluations, it raises ethical concerns regarding the monetization of human life. Assigning economic value to life years lost due to cancer may be perceived as reductionist, yet it provides a standardized framework for comparing public health burdens and informing policy decisions.

The joinpoint regression model, developed by the US National Cancer Institute, estimates temporal trends for disease burden and economic loss. In practice, we began with a model containing no joinpoints (i.e., a single linear segment) and sequentially tested whether adding a joinpoint improved the model fit significantly. The maximum amount of joinpoints allowed is 4 joinpoints. At each step, a Monte Carlo permutation test was performed to assess if the introduction of an additional joinpoint led to a statistically significant improvement in model fit (indicating a true change in trend). We estimated the Annual Percent Change (APC) for each segment and the Average Annual Percent Change (AAPC) for the overall period. This permutation approach involves generating numerous random rearrangements of the data to establish a null distribution of the test statistic under the assumption of no additional joinpoints; the observed change in fit is then compared against this reference distribution. If adding a joinpoint yielded a reduction in residual error that was unlikely under the null hypothesis (for example,* p* < 0.05), that joinpoint was incorporated into the model. If the improvement was not statistically significant, the joinpoint was not added (and no further joinpoints were tested). We continued this iterative process until no further significant joinpoints could be identified (or until a preset maximum number of joinpoints was reached), ensuring that each joinpoint included in the final model corresponded to a statistically significant change in the trend. By using Monte Carlo permutation tests for both model selection and hypothesis testing in this manner, our approach adhered to a robust, data-driven methodology and avoided reliance on information criteria (AIC or BIC) for determining the number of trend changes [16].

## Results

### Global temporal trend

**Fig. 1:**
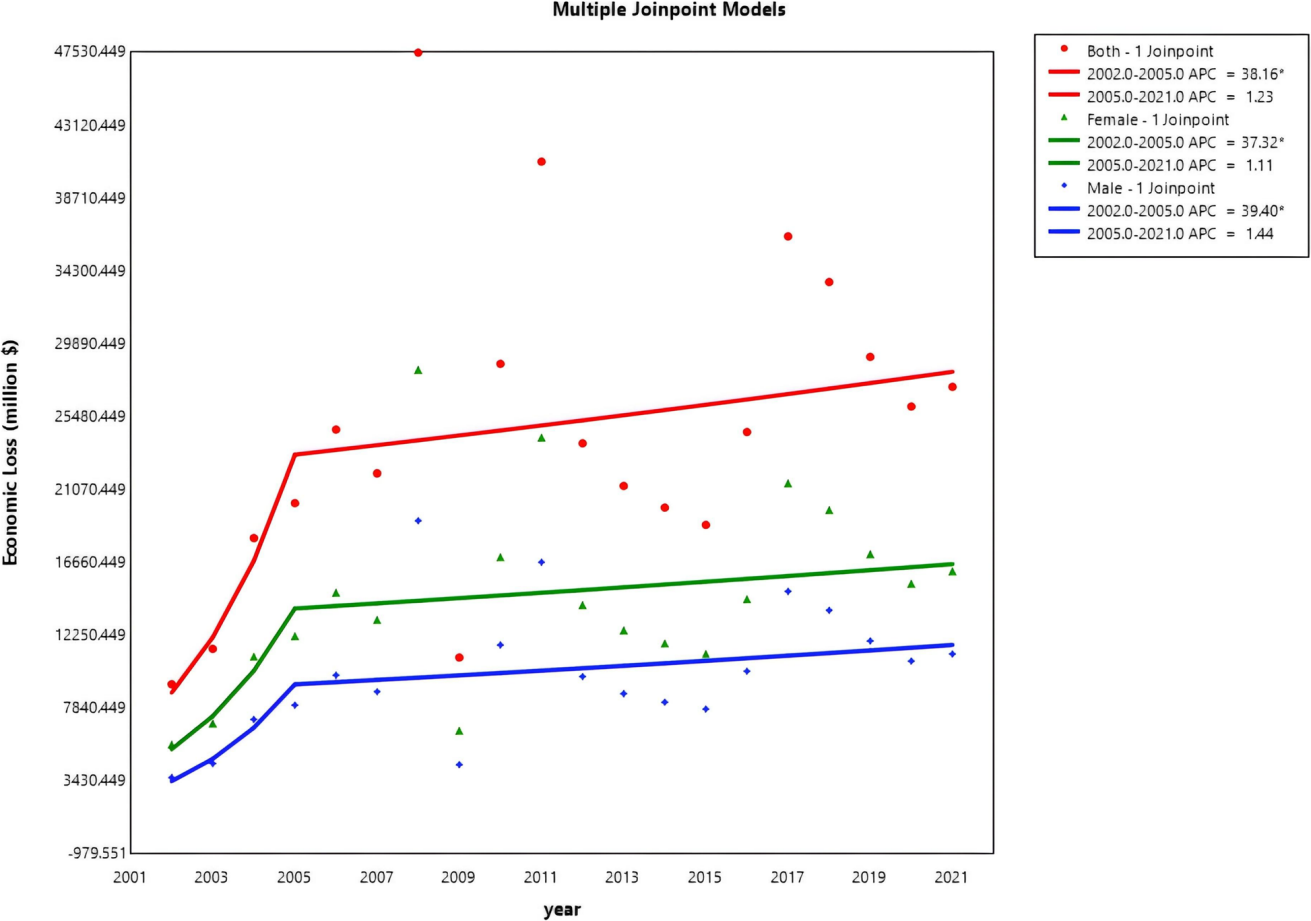
Joinpoint regression for general trends in economic loss

The specific disease burden indicated by age-standardized deaths, DALYs, and Economic Loss calculated from total DALYs is shown in the appendix. On the global level, both the age-standardized death rate, DALYs rate, and total economic loss show a generally increasing pattern. eTable 1 shows that while the death rate and DALYs rate showed a decreasing trend in the first period, especially for females, they bounded up in the following years and reached 4.18 per 100,000 for death (UI: 1.71–6.8) and 102.17 per 100,000 for DALYs (UI: 43.24–165.02) (eTable 6) (Fig. S1, S2, and S3). The economic loss showed increasing but insignificant results during the second period, as there was more fluctuation in the second stage (Fig. 1). All the measures have a breakpoint from around 2005 to 2007.

### Sex–cancer type specific

eTable 6 indicates that females have a higher burden in age-standardized DALYs and death rates, and Fig. 1 shows the higher burden of females in economic loss than males. However, eTable 2 witnessed a shrinking gender ratio (1.55 to 1.45) female-to-male ratio of economic loss (from 1.55 to 1.45) globally and for most cancers (we excluded cancers that only females will suffer). Despite thyroid, pancreatic, gallbladder, and biliary tract cancers, males had a more significant loss in other types of cancers. Thyroid cancer had the highest gender gap (1.77 to 1.55) regarding more considerable loss in women, and kidney cancer showed the most significant relative loss in men (0.55 to 0.52), where the gap even grew larger. Pancreatic cancer decreased the most (2.08 to 1.32), whereas there was little change for non-Hodgkin lymphoma (stayed at 0.79 in 2002 and 2021).

eTable 3 shows the temporal trends for different types of cancer. All types of cancers increased in the economic loss. Pancreatic cancer (AAPC: 10.47*, CI: 8.01–13.51) and liver cancer (AAPC: 8.08*, CI: 5.77–10.35) had the fastest growing trend, and their age-standardized deaths also showed a surge during this period (eTable 6), while leukemia is the slowest (AAPC: 5.08, CI: 2.85–7.27) and even declined slightly in the second period (APC: −0.07, CI: −2.97–1.85).

**Fig. 2:**
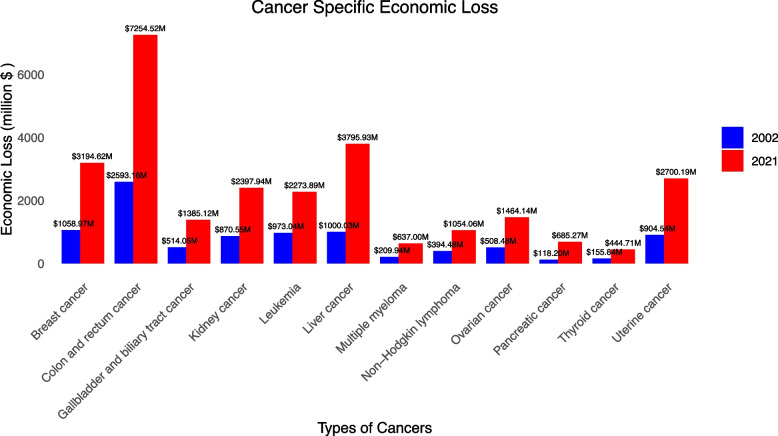
Cancer-specific economic loss

**Fig. 3:**
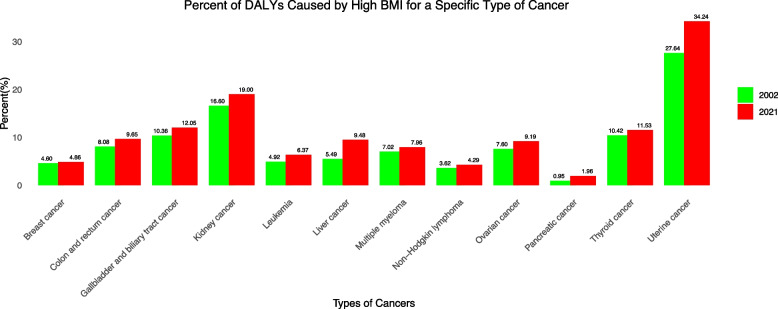
Percent of DALYs caused by high BMI for a specific type of cancer

Figure 2 delineates the economic loss for each type of cancer in 2002 and 2021. Colon and rectum cancer always had the highest loss ($2593.159 million, UI: 1109.04–4119.61, to $7294.52 million, UI: 3134.75–11,511.13), while Thyroid cancer had the lowest loss ($155.84 M in 2002, UI: 118.28–197.43, and $444.71 M in 2021, UI: 335.10–566.78). Figure 3 shows the percentage of high-BMI-caused specific cancer among all these specific types of cancer. Uterine cancer took the highest proportion (27.64% to 34.24%), whereas pancreatic cancer constituted the most minor (0.95% to 1.96%). All types of cancers underwent a growing proportion.

### Income tier and region-specific

**Fig. 4:**
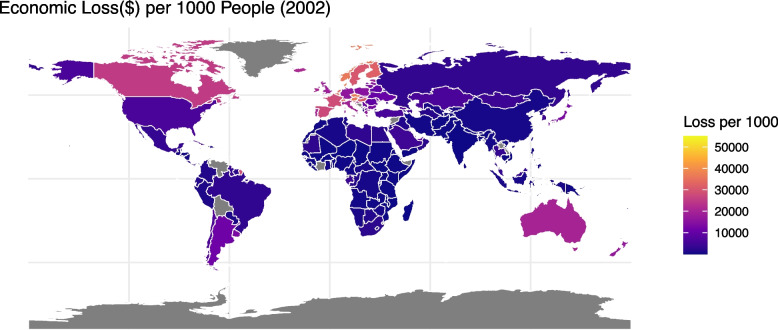
Economic loss per 1000 people in 2002

**Fig. 5:**
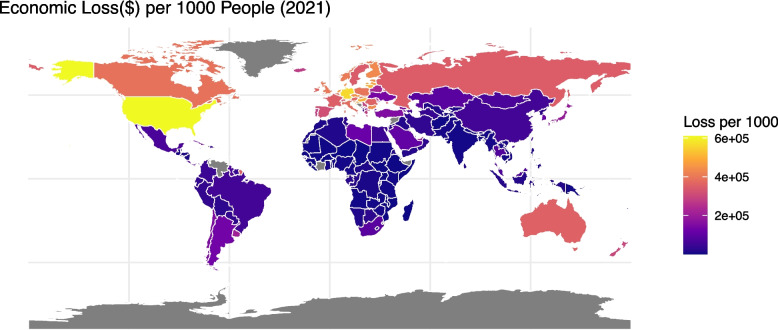
Economic loss per 1000 people in 2001

Figures 4 and 5 compare the economic loss per 1000 population in different countries due to HBCCs in 2002 and 2021. Nineteen countries have missing data due to a lack of information about economic indexes. The whole world increased in value dramatically. The USA and Russia increased markedly, with only $6433.103(UI: 2512.728–10,638.18) and $3199.299 (UI: 1375.916–5030.125) in 2002 respectively (Fig. S4), and $608,353.2(UI: 247,916.8–986,420) and $349,932.3 (UI: 142,777.5–563,140.1) in 2021 (Fig. S5). Europe and Australia were always among the most serious. Africa and the Middle East have generally suffered the least from HBCCs except UAE ($10,975.21 in 2002, UI: 5183.722–17,668.69, and $218,447.8 in 2021, UI: 95,207.2–350,872.5). Most Asian countries in China are always not very high in the economic loss of HBCCs, while Japan is relatively higher than other countries in Asia ($13,406.37 in 2002, UI: 6189.948–21,091.64, and $190,713.3 in 2021, UI: 81,530.76–300,676.7). Supplementary Fig. 3 and Supplementary Fig. 4 compare the age-standardized DALYs in different countries in 2002 and 2021. It is generally an increasing trend, and higher-income countries show a higher burden. However, in contrast to the economic loss, Mongolia indicated very high losses in age-standardized DALYs (295.90 in 2002 and 326.23 in 2021), while higher-income countries like the USA showed a slight decreasing trend (210.34 in 2002 and 199.29 in 2021).

**Fig. 6:**
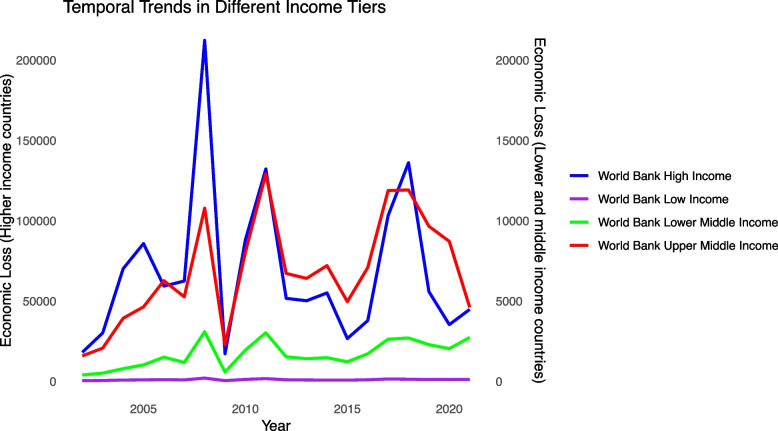
Temporal trends in different income tiers

For different income tiers, it is the high-income group that suffers the most from HBCCs, and there is a massive gap between low and middle-income countries and high-income countries both for the epidemiologic burden and the economic loss (eTable 6), but their growing patterns are similar. Fluctuations are ubiquitous in tiers except for the low-income group, with high-income countries undulating the most. All the tiers witnessed a peak at around 2008 (Fig. 6). Also, eTable 2 illustrates the smaller gender gap in higher-income countries, though the gap is shrinking in all the income tiers. eTable 4 shows that low and middle-income countries generally experienced a significant increase during the time frame. In contrast, high-income countries have a much higher growth rate in the first stage (APC: 119.75*), but show a trend not significant in economic burden in the second stage (APC: − 2.15).

eTable 5 shows the specific type of cancer that caused the highest and lowest loss in each income group. In 2002, CRC was the highest loss for high, upper–middle, and lower-middle-income groups; liver cancer was for low-income countries. Except for high-income countries, where Thyroid Cancer was the least, Pancreatic cancer contributed the least for the other three income tiers. In 2021, CRC had become the highest in the whole world, but Thyroid cancer had become the least for not only high–income but also upper-middle-income countries.

## Discussion

### Summary of major findings

On the global level, DALYs and deaths showed an increasing trend from 2002 to 2021. The economic loss from total DALYs increased by 193%. This is not only related to the growth of population size and the aging population but also to the exacerbating situation of obesity. According to the World Health Organization, the prevalence of obesity more than doubled between 1997 and 2022 [17]. Modern people’s lifestyles have changed significantly with improved living conditions. In addition, the rapid growth of economic loss in the early period is likely due to a combination of rising obesity rates and significant economic growth (increasing the VSL) during those years. After an initial surge in the early 2000s, the increase in economic loss slowed after 2005, while death and DALYs have shown upward trends. This bifurcation is due greatly to the undulation in the value of life caused by the skittish financial market largely driven by fluctuations in the applied discount rate (real interest rate) over time, which resulted in corresponding changes in the value of a statistical life and thus the economic loss. Colon and rectum cancer (CRC) show the highest economic loss ($2593.159 million, UI: 1109.04–4119.61, to $7294.52 million, UI: 3134.75–11,511.13), with pancreatic (AAPC: 10.47*, CI: 8.01–13.51) and liver cancer (AAPC: 8.08*, CI: 5.77–10.35) being the fastest–growing cause. The surge in these two types of cancers can be attributed to their amounting mortality. For pancreatic cancer, the age-standardized death rate was 0.051 (UI: − 0.04 ~ 0.20) in 2002 and 0.106 (UI: − 0.029 ~ 0.308) in 2021; for liver cancer, it was 0.366 (UI: 0.152 ~ 0.593) in 2002 and 0.534 (UI: 0.215 ~ 0.901) in 2021. But both measures for loss in pancreatic cancer are at a very low level, signaling the loss in liver cancer might be more critical.

Despite the shrinkage in the gender ratio of the economic loss of cancer due to high BMI (female/male: 1.55 to 1.45) and the fact that males had a higher health burden in most types of cancers, females always had a higher economic loss for total cancer. This is mainly for three reasons. First, the health burden is expressed using age-standardized indices, which exclude the effects of population growth and demographic shifts, thereby providing a more stable measure of disease burden over time. In contrast, the economic burden is calculated based on total DALYs, as this metric must account for the influence of population changes and age structure variations to accurately reflect the societal cost of disease. Then, the difference in body composition between females and males is associated with different risks of cancers. Males have higher muscle mass than females, but low muscle mass is linked with a higher risk of recurrence, mortality, surgical complications, and treatment-related toxicities [18]. Aside from that, a higher proportion of fat stored in women’s abdomen is associated with an increased risk of diseases like esophageal squamous cell carcinoma [19]. Second, obesity is more prevalent in women than in men in most countries [20]. From a medical perspective, obesity is associated with female-specific factors such as estrogen levels, basal metabolic rate, menstrual cycle, and menopause [21]. Besides that, compared to men, women are also more susceptible to emotions [22], undertaking more roles in society, which leads to a higher possibility of seeking comfort food and being less active in sports, increasing the risk of fat accumulation [23], especially during pregnancy. Another driving force is the type of cancer they suffer. High BMI is strongly related to cancers that are specific to women; for example, Uterine Cancer takes the most significant proportion that high BMI causes. In addition, obesity may also lead to changes in estrogen levels, which can increase the risk of gynecological tumors like ovarian cancer [24].

It is also important to consider socioeconomic factors: our analysis used a country’s average VSL for both sexes, and thus did not account for gender disparities in income, labor participation, or healthcare access that could affect economic loss. Women generally have lower wages than men and lower labor force participation rates [25], which may influence the economic impact of a given health loss. Additionally, gender-based differences in access to healthcare can affect outcomes—in many societies, gender inequality poses barriers for women in obtaining health information and critical services [26], potentially leading to later cancer detection or reduced treatment opportunities. These factors could partially explain why women bear a higher economic burden despite men’s greater health burden in most types of cancers, and we acknowledge that further investigation into gender-specific economic effects is needed.

The cancer burden for all measures positively correlates with the country’s income level. Higher-income countries have higher health and economic burdens in HBCCs because obesity is more prevalent in the developed world [27]. In addition, because our economic burden metric (VSL-based) scales with income, higher-income countries incur a greater monetary loss per unit of health loss, which also contributes substantially to the observed gradient between rich and poor countries. However, there are also differences within high-income countries. The DALYs and economic loss are much higher in the USA than in other developed countries. In addition to the larger population in the USA, this is partly because the obesity problem is relatively severe in the USA but not so critical in countries like the UK less severe in some other high-income countries. UAE is low in economic loss for it has a smaller population, but it still has very high disease burden given its age-standardized DALYs at 268.81 (UI: 114.46–436.41) in 2021, while the USA is 199.29 (UI: 83.07–319.71). Aside from environmental pollution due to urbanization and the larger population in the Northern World, which exacerbate the situation for all types of cancers, the surge in disease burden of HBCCs in the USA, China, and Russia is also related to cultural backgrounds, diet habits, and changes in the social environment. Economic growth and the rise in wages during the past 20 years, especially in China, increased the value of statistical life, which enlarges the financial economic loss.

Higher-income countries showed a decreasing trend in health burden (age-standardized death rate and DALYs rate), but other income tiers are still increasing, where the influence from the population change is excluded by standardizing to per 100,000 population data, possibly due to the enhanced treatment level and better affordability in the developed world. A key driver of the increasing economic burden in high-income countries, despite declining health burdens, is the method of burden quantification. While health burden is measured using age-standardized rates, which adjust for demographic differences and exclude the influence of total population and aging, economic burden is calculated based on total DALYs, reflecting absolute disease burden across all age groups. High-income countries tend to have larger absolute populations of individuals with obesity-related cancers, leading to higher total DALYs, even if their age-standardized rates are declining. Moreover, higher life expectancy in high-income countries results in more years of life lost (YLL) per obesity-related death, further increasing total DALYs and, consequently, economic loss estimates [28]. This distinction explains why economic loss continues to rise in high-income settings despite improvements in medical treatment and reductions in age-adjusted mortality rates. Additionally, while obesity prevalence has plateaued or slightly declined in some high-income countries, it remains significantly higher than in many low- and middle-income countries, sustaining a substantial total burden. In contrast, in low- and middle-income countries, obesity rates are still rising, but their age-standardized DALY rates remain higher due to a younger population structure, which means fewer life-years lost per death compared to high-income settings.

High-income countries fluctuate more in terms of economic burdens but not health burdens, partly explained by their reliance on the skittish financial market, which disturbs the value of work age. Financial factors more significantly impact high-income countries and upper-middle-income countries. The 2008 global financial crisis, triggered by the collapse of Lehman Brothers and the subsequent subprime mortgage crisis, had profound economic repercussions. This shock influenced the valuation of life during that period, as financial instability reshaped economic priorities and resource allocation. In 2015, global financial markets experienced significant volatility, including a sharp summer downturn in China’s stock market and the Federal Reserve’s end to its prolonged zero-interest-rate policy. These fluctuations affected household wealth effects and consumption tendencies, alongside potential impacts from slowing growth in emerging economies and the energy market. While these factors may have influenced perceptions and valuations of life, the shock was less severe than in 2008.

Beyond differences in obesity prevalence, income disparity between high-income countries (HICs) and low- and middle-income countries (LMICs) likely plays a more substantial role in explaining the gradient in economic burden. The economic burden is directly influenced by the value of a statistical life (VSL), which scales with national income levels. As a result, HICs experience significantly higher economic losses simply because lost years of life are valued more in monetary terms. Additionally, higher wages and greater labor market participation rates in HICs amplify the economic implications of premature mortality and morbidity, further widening the economic burden gap between income tiers. Future research could explore this dynamic in greater depth by quantifying the relative contributions of obesity prevalence versus income disparity in shaping economic burden trends.

The gender gap shrank in 3 income tiers but witnessed a minor growth in low-income countries, possibly because the gender disparity is less significant for the developed world. CRC contributed the most to the loss in all the tiers as it is the third-largest cause of mortality and has the second-largest prevalence among all cancers [7], and pancreatic cancer contributed the least. However, in upper-middle-income and high-income countries, thyroid cancer took the place of being the most minor contributor to the economic burden in 2021.

### Policy implications

Our findings highlight that governments worldwide, especially those in developed countries and emerging economies with rapid economic growth, should focus more on curbing the growing obesity problem. This requires policy changes, cost-effective obesity prevention programs, food taxation policies, and public health campaigns that promote healthier lifestyles. For instance, taxing sugar-sweetened beverages and high-calorie processed foods has been shown to reduce consumption and lower obesity rates [29]. In addition, workplace wellness programs that incentivize employees to engage in physical activity and adopt healthier eating habits could contribute to long-term reductions in obesity prevalence.

For higher health and economic burdens in females, more emphasis should be put on curbing the fat accumulation, especially during post-pregnancy, and measures should be taken to improve the targeted group, like inner-city women’s adherence to cancer screening to intervene in the incidence of cancer earlier [30] adherence to cancer screening among targeted high-risk groups (e.g., inner-city women). Further interdisciplinary research in labor, health, and family economics is also required.

Cost-effectiveness analyses suggest that reducing obesity prevalence by even 5–10% could yield significant savings in healthcare costs. Estimates indicate that for every 5% reduction in obesity rates, national healthcare expenditures related to obesity and obesity-related cancers could decline by up to $187 billion annually, depending on country-specific healthcare structures and costs. Such savings could be reinvested in public health initiatives aimed at sustainable obesity reduction.

Not only specific health insurance programs targeted at females but also legislative instruments that promote women’s employment stability and workplace flexibility. In addition, friction costs that their employers have encountered when seeking a replacement in labor are also expected to be considered, and policy benefits should be tilted toward companies with a large proportion of overweight women to prevent gender disparity in the job-hunting market [31]. Targeting specific types of cancer, CRC is expected to get the most attention from academic research as the underlying mechanisms, like obesity and CRC, remain unclear. Liver cancer is also supposed to be focused on as it shows a fast-growing trend, and more research should be conducted to discuss measures to tackle its increasing mortality rate.

### Limitations

Our research suffers from two types of limitations. First, we share the limitations of the GBD 2021 Database. Underreporting might happen in countries with poor access to healthcare [9], and cut points for high BMI vary by race and ethnicity [32]. Aside from that, we cannot compare the disease burden of HBCCs between overweight and obesity as a specific figure for BMI is not provided in this dataset. Besides, the Value of a Statistical Life Approach cannot account for the economic adjustment mechanism as the substitution of labor lost due to illness by capital or other workers is not quantified [33]. In addition, because our economic burden estimates are tied to macroeconomic values (income and VSL), they may reflect short-term financial volatility that does not mirror actual health changes. This could lead to large year-to-year swings in economic loss (for instance, the sharp drop around the 2008 financial crisis), so caution is needed in interpretation; future work might consider smoothing techniques or alternative models to address such fluctuations. With regard to joinpoint regression, we acknowledge that the specific joinpoint locations might vary with different model specifications or if using a different approach.

## Conclusions

This study is based on data from the GBD 2021 database, which was used to extract information on total disability-adjusted life Years (DALYs), morbidity, and mortality associated with HBCCs worldwide from 2002 to 2021. To estimate the economic loss for each cancer in each group, we employed the Value of Statistical Life Approach (VSLA) that incorporates a willingness-to-pay metric and estimates the financial loss due to total DALYs. A joinpoint regression analysis was utilized to capture the temporal trends.

The disease burden of HBCCs is concerning and positively correlated with income level. Economic loss and health burdens are, in most cases, similar in growth patterns but sometimes differ as the monetary value of working age varies in different income tiers. Females always have higher burdens due to the prevalence of obesity and the types of cancers they suffer. Certain types of cancers contribute much more to economic loss, and pancreatic and liver cancer are the fastest-growing causes due to the high mortality. These findings necessitate further investigation of government subsidy policies and intervention programs.

## Supplementary Information

Additional File 1: Figures S1-S5. Fig. S1. Trends for Age-standardized Death rate. Fig. S2. Trends for Age-standardized DALYs rate. Fig. S3. Trends for Economic Loss in Certain Type of Cancer. Fig. S4. Worldwide Hotspots in Age-standardized DALYs due to HBCCs in 2002. Fig. S5. Worldwide Hotspots in Age-standardized DALYs due to HBCCs in 2021.

eTable 1 Global Temporal Trend. eTable 2 Gender Ratio in Economic Loss. eTable 3 Temporal Trends of Different Types of Cancer. eTable 4 Temporal Trends for Different Income Tiers. eTable 5 Types of Cancer with the Highest and Lowest Loss. eTable 6 Health and Economic Burden.

## Data Availability

No datasets were generated or analysed during the current study.

## Abbreviations

APC: Annual percentage change
AAPC: Average APC
CODEm: Cause of Death Ensemble model
CRC: Colon and rectum cancer
DALYs: Disability-adjusted life years
GBD: Global Burden of Disease
GNI: Gross National Income
HBCCs: High BMI-caused cancers
LE: Life expectancy
MA: Mid-age point
UIs: Uncertainty intervals
VSLA: Value of Statistical Life Approach
VSLY: Value of Statistical Life per Year
