# Supplementary material for: Economic burden attributable to high BMI-caused cancers: a global level analysis between 2002 and 2021
Source: BMC Med. 2025 May 28;23:297. doi: 10.1186/s12916-025-04109-8 (PMC12121180; doi:10.1186/s12916-025-04109-8)
Supplement: Supplementary file 1 — Additional File 1: Figures S1-S5. Fig. S1. Trends for Age-standardized Death rate. Fig. S2. Trends for Age-standardized DALYs rate. Fig. S3. Trends for Economic Loss in Certain Type of Cancer. Fig. S4. Worldwide Hotspots in Age-standardized DALYs due to HBCCs in 2002. Fig. S5. Worldwide Hotspots in Age-standardized DALYs due to HBCCs in 2021. [file 12916_2025_4109_MOESM1_ESM.docx]

**Additional File 1: Figures S1-S5.**

**Fig. S1.** Trends for Age-standardized Death rate

**Fig. S2.** Trends for Age-standardized DALYs rate

**Fig. S3.** Trends for Economic Loss in Certain Type of Cancer

**Fig. S4.** Worldwide Hotspots in Age-standardized DALYs due to HBCCs in 2002

**Fig. S5.** Worldwide Hotspots in Age-standardized DALYs due to HBCCs in 2021

**
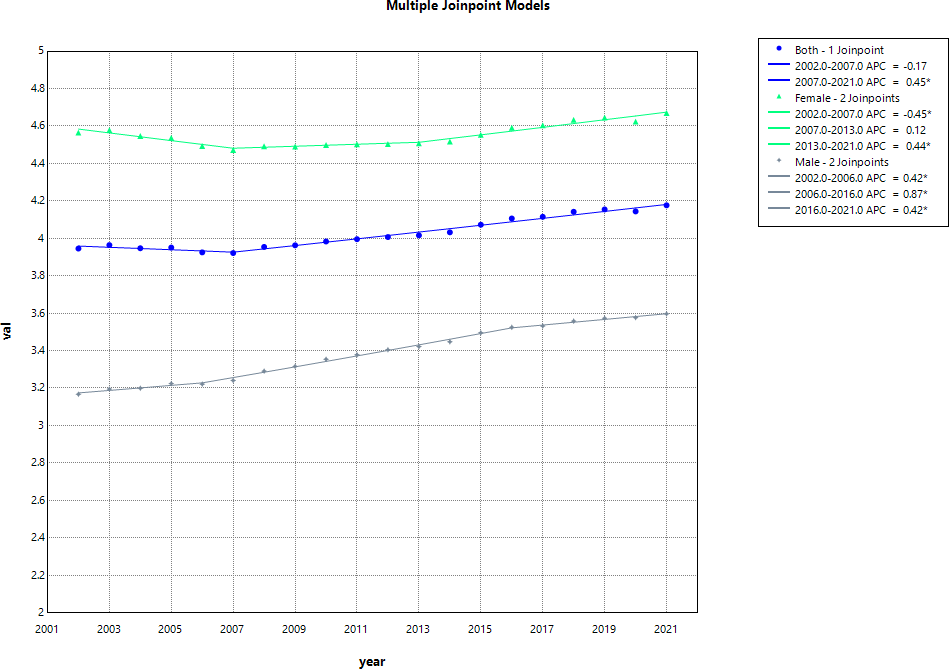
Fig. S1.** Trends for Age-standardized Death rate

**
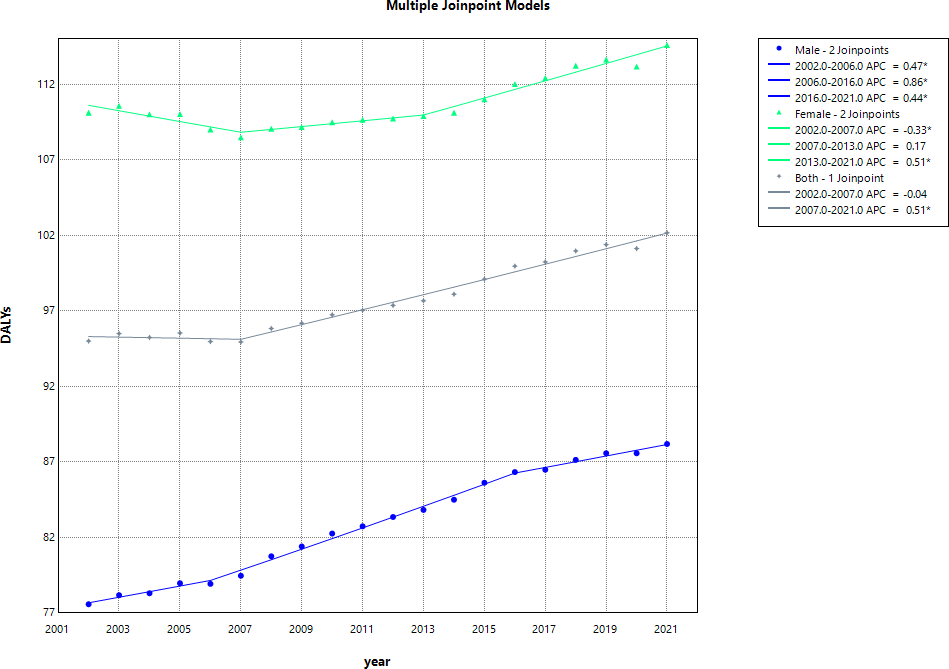
Fig. S2.** Trends for Age-standardized DALYs rate

**Fig. S3.** Trends for Economic Loss in Certain Type of Cancer

| 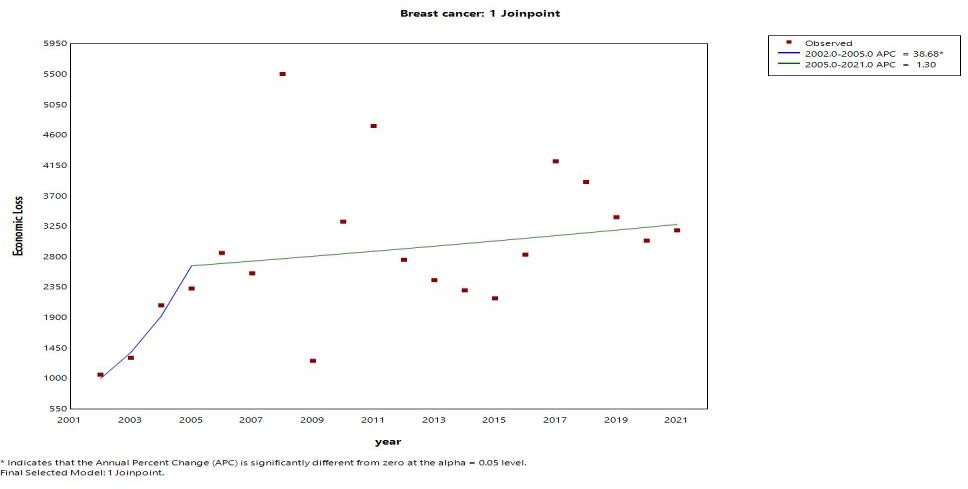 | 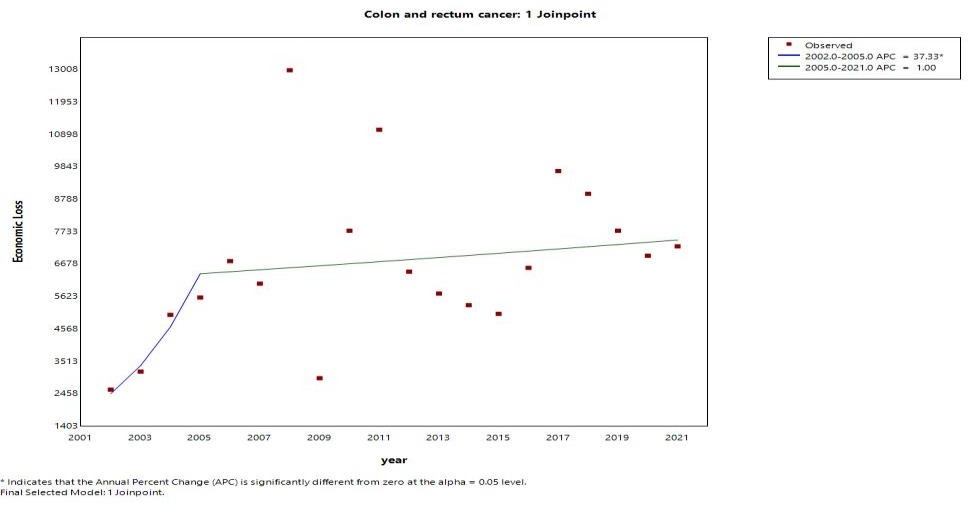 |
| --- | --- |
| 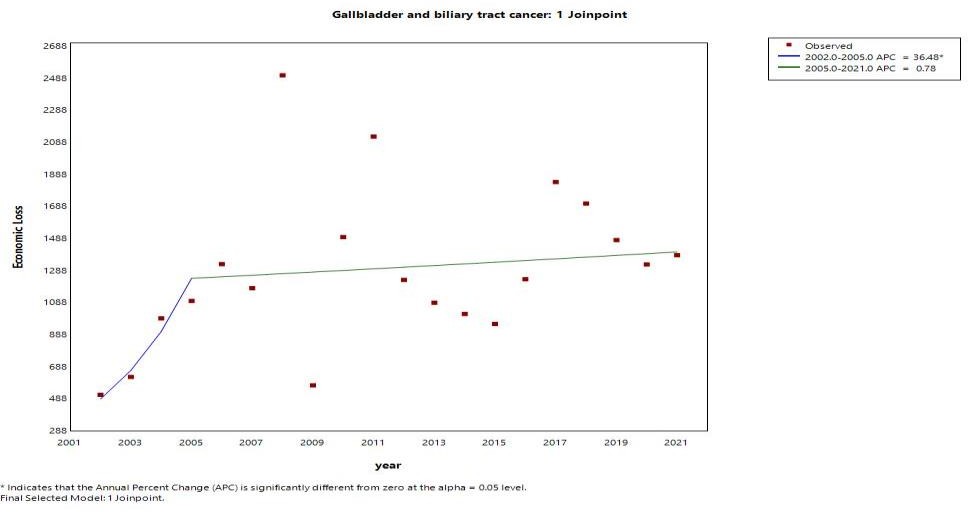 | 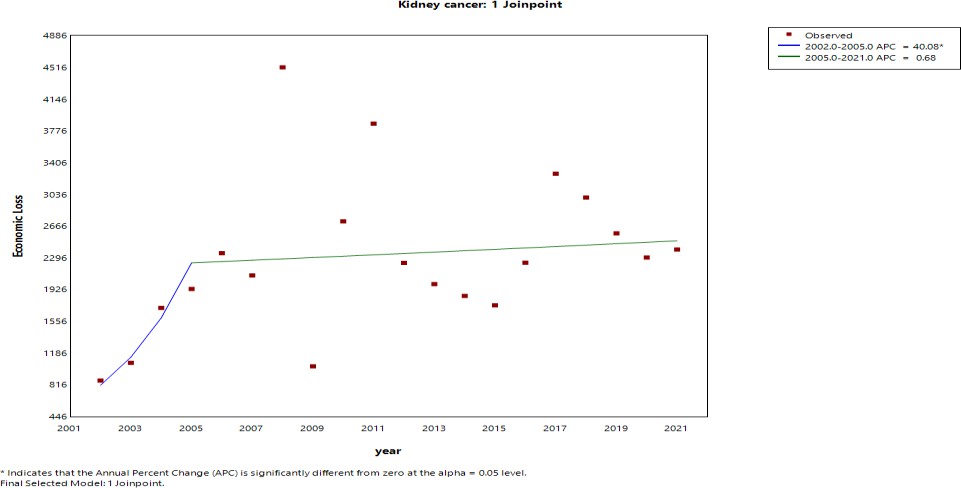 |

| 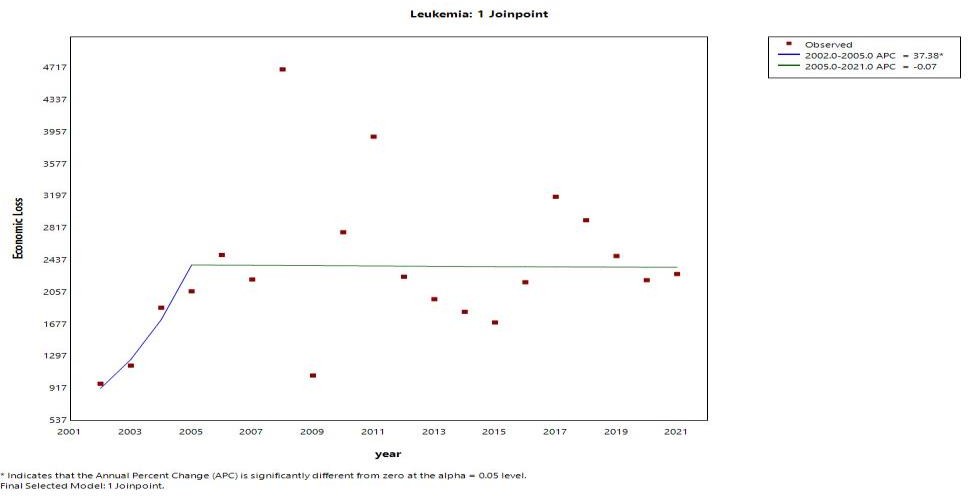 | 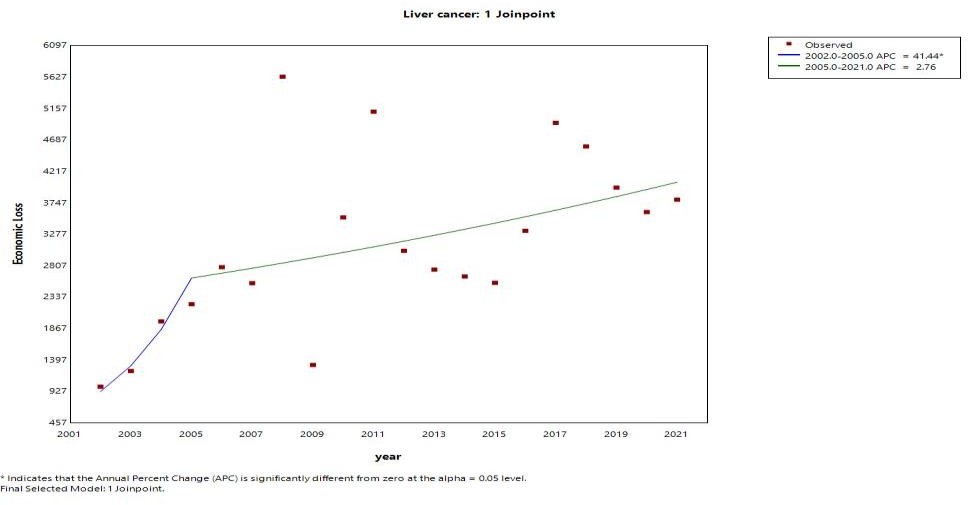 |
| --- | --- |
| 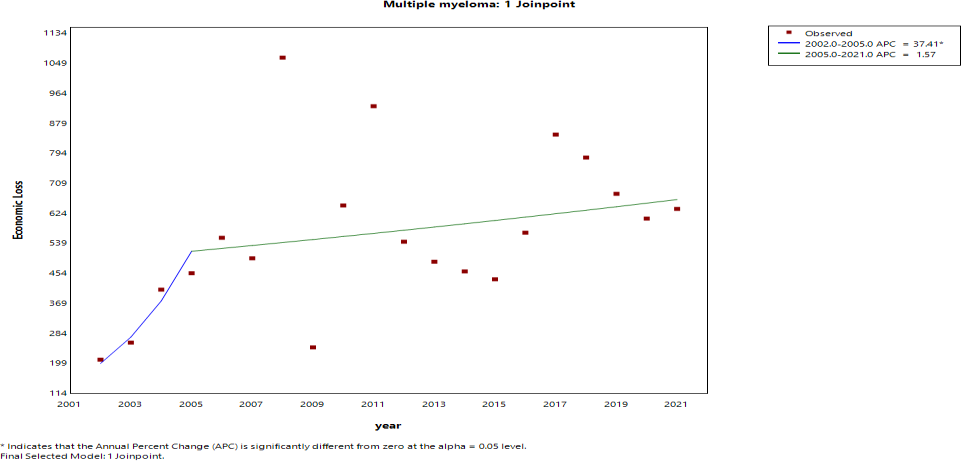 | 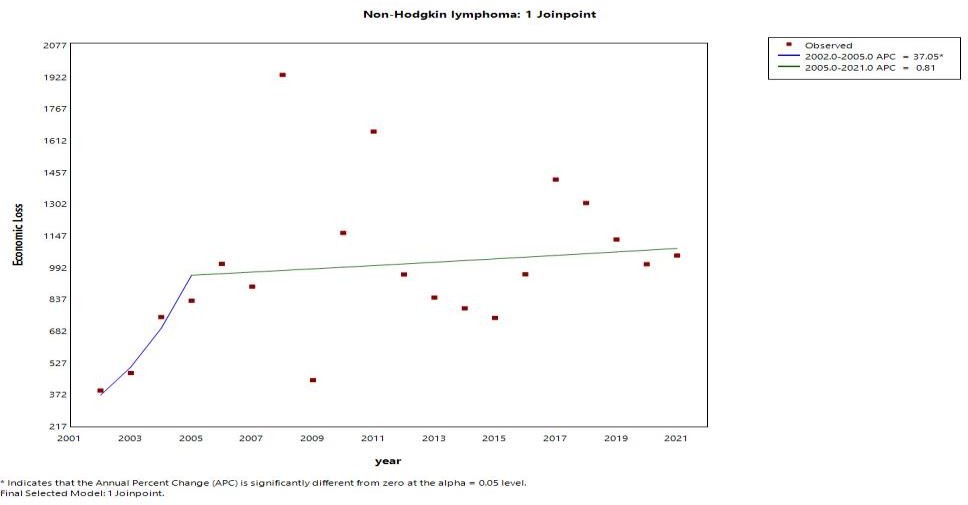 |

| 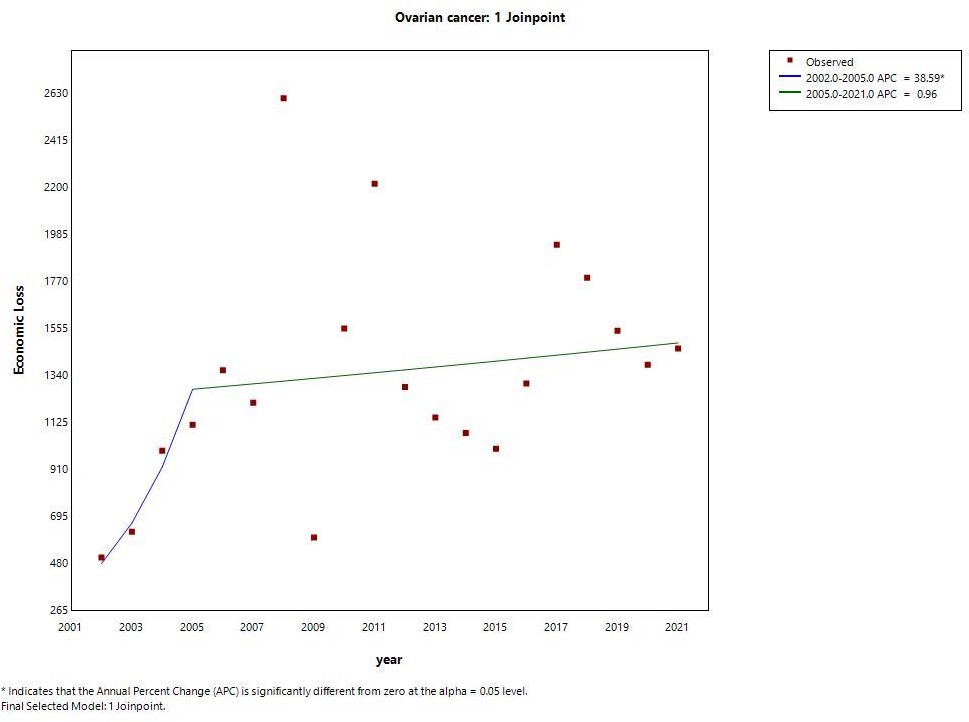 | 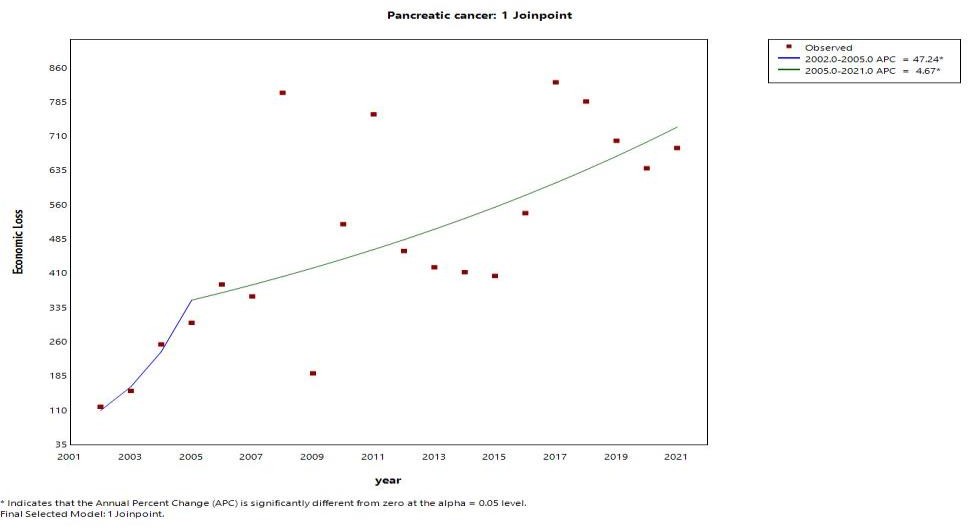 |
| --- | --- |
| 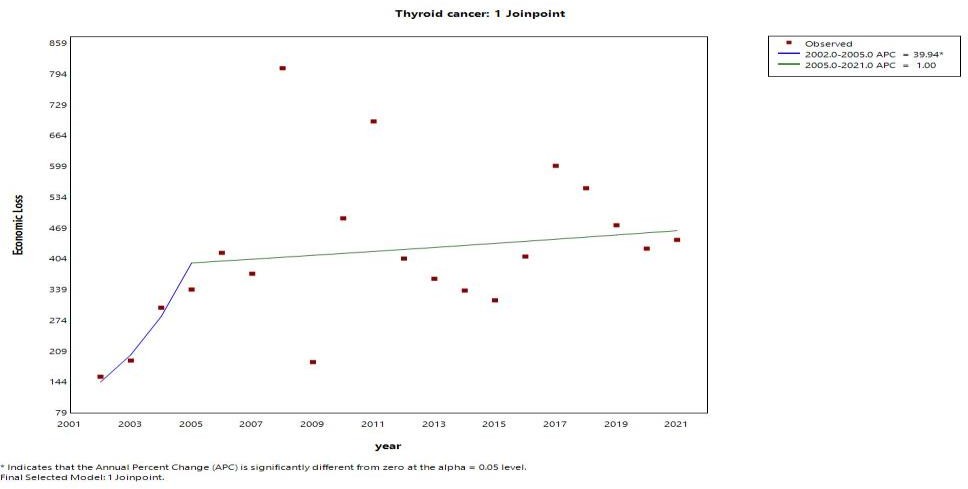 | 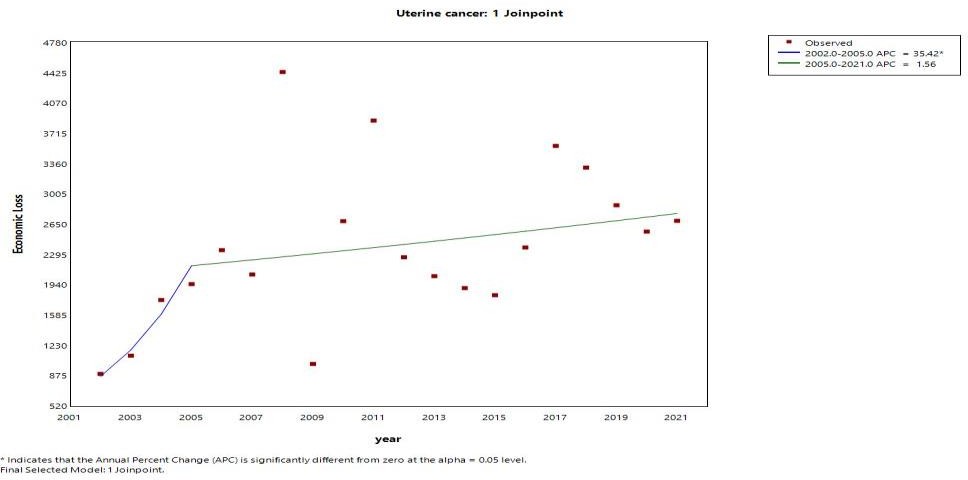 |

**Fig. S4.** Worldwide Hotspots in Age-standardized DALYs due to HBCCs in 2002


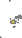

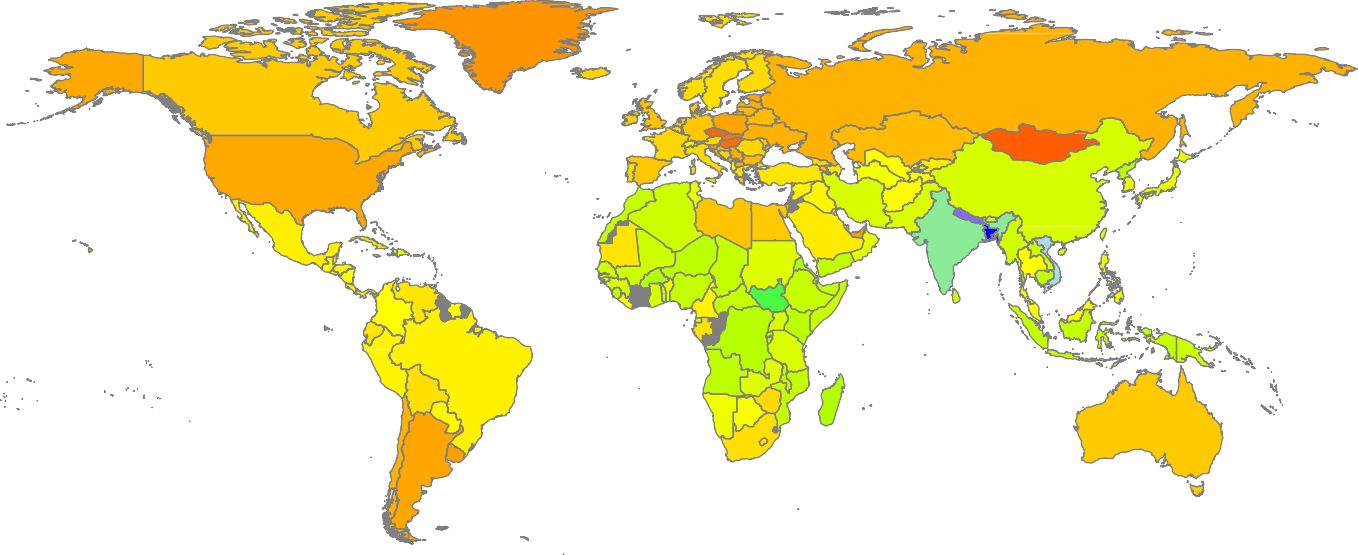

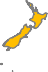

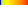


DALYs

50

100

150

200

250

300

**Fig. S5.** Worldwide Hotspots in Age-standardized DALYs due to HBCCs in 2021


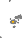

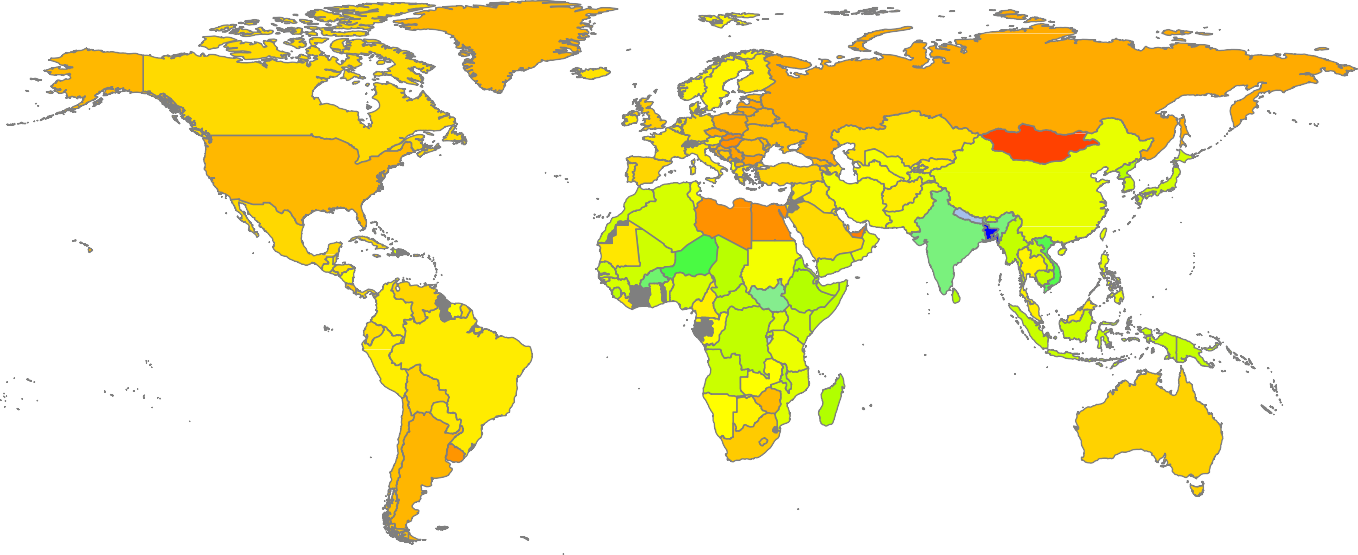

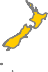

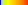


DALYs

50

100

150

200

250

300

350
