# Supplementary material for: Economic burden attributable to high BMI-caused cancers: a global level analysis between 2002 and 2021
Source: BMC Med. 2025 May 28;23:297. doi: 10.1186/s12916-025-04109-8 (PMC12121180; doi:10.1186/s12916-025-04109-8)
Supplement: Supplementary file 2 — eTable 1 Global Temporal Trend. eTable 2 Gender Ratio in Economic Loss. eTable 3 Temporal Trends of Different Types of Cancer. eTable 4 Temporal Trends for Different Income Tiers. eTable 5 Types of Cancer with the Highest and Lowest Loss. eTable 6 Health and Economic Burden. [file 12916_2025_4109_MOESM2_ESM.docx]

# **eTable 1 Global Temporal Trend**

|  |  |  |  | **Joinpoint Analysis (2002–2021)** | | | | | | | | |
| --- | --- | --- | --- | --- | --- | --- | --- | --- | --- | --- | --- | --- |
| **Index** | **Sex** | **AAPC** | | Trend 1 | | | Trend 2 | | | Trend 3 | | |
|  |  | **value** | **CI** | Year | APC | CI | Year | APC | CI | Year | APC | CI |
| Death Rate | Both | 0.29* | 0.25–0.33 | 2002–2007 | -0.17 | -0.69–0.06 | 2007–2021 | 0.45* | 0.40–0.52 |  |  |  |
| Death Rate | Female | 0.10* | 0.06–0.15 | 2002–2007 | -0.45* | -0.93– -0.22 | 2007–2013 | 0.12 | -0.27–0.34 | 2013–2021 | 0.44* | 0.33–0.80 |
| Death Rate | Male | 0.66* | 0.62–0.70 | 2002–2006 | 0.42* | 0.02–0.62 | 2006–2016 | 0.87* | 0.82–1.00 | 2016–2021 | 0.42* | 0.22–0.57 |
| DALYs Rate | Both | 0.37* | 0.33–0.41 | 2002–2007 | -0.04 | -0.60–0.19 | 2007–2021 | 0.51* | 0.46–0.59 |  |  |  |
| DALYs Rate | Female | 0.18* | 0.15–0.22 | 2002–2007 | -0.33* | -0.70– -0.15 | 2007–2013 | 0.18 | -0.08–0.39 | 2013–2021 | 0.51 | 0.42–0.78 |
| DALYs Rate | Male | 0.67* | 0.63–0.71 | 2002–2006 | 0.47 | 0.04–0.70 | 2006–2016 | 0.86 | 0.81–1.14 | 2016–2021 | 0.44 | 0.17–0.60 |
| Economic Loss | Both | 6.32* | 4.04–8.57 | 2002–2005 | 38.16* | 15.59–87.23 | 2005–2021 | 1.23 | -1.85–3.16 |  |  |  |
| Economic Loss | Female | 6.12* | 3.81–8.40 | 2002–2005 | 37.31* | 14.78–86.44 | 2005–2021 | 1.11 | -2.09–3.05 |  |  |  |
| Economic Loss | Male | 6.66* | 4.42–8.88 | 2002–2005 | 39.40* | 17.00–88.53 | 2005–2021 | 1.44 | -1.53–3.38 |  |  |  |

Note: * indicates that the APC or AAPC significantly differs from 0 at the alpha=0.05 level, and CI means the 95% confidence interval.

# **eTable 2 Gender Ratio in Economic Loss**

| **Region/Cancer Type** | **2002 Gender Ratio** | **2021 Gender Ratio** |
| --- | --- | --- |
| ***Region*** |  |  |
| Global | 1.55 | 1.45 |
| World Bank High Income | 1.40 | 1.31 |
| World Bank Upper Middle Income | 1.63 | 1.41 |
| World Bank Lower Middle Income | 1.90 | 1.91 |
| World Bank Low Income | 1.72 | 1.65 |
| ***Type of Cancer*** |  |  |
| Colon and rectum cancer | 0.96 | 0.86 |
| Liver cancer | 0.55 | 0.54 |
| Leukemia | 0.87 | 0.83 |
| Thyroid cancer | 1.77 | 1.55 |
| Pancreatic cancer | 2.08 | 1.32 |
| Non–Hodgkin lymphoma | 0.79 | 0.79 |
| Multiple myeloma | 0.99 | 0.93 |
| Gallbladder and biliary tract cancer | 1.73 | 1.47 |
| Kidney cancer | 0.55 | 0.52 |

**Note:** For specific types of cancer, types that only females suffer are excluded—Breast cancer, ovarian cancer and uterine cancer. The types here are only those with both male and female patients.

# **eTable 3 Temporal Trends of Different Types of Cancer**

|  |  |  | Joinpoint Analysis (2002–2021) | | | | | |
| --- | --- | --- | --- | --- | --- | --- | --- | --- |
| **Type of Cancer** | **AAPC** | | Trend 1 | | | Trend 2 | | |
|  | **Value** | **CI** | Year | APC | CI | Year | APC | CI |
| Colon and rectum cancer | 6.02* | 3.73–8.29 | 2002–2005 | 37.33* | 14.72–18.36 | 2005–2021 | 1.00 | -2.16–2.93 |
| Uterine cancer | 6.28* | 3.91–8.60 | 2002–2005 | 35.42* | 13.01–83.31 | 2005–2021 | 1.56 | -1.89–3.49 |
| Liver cancer | 8.08* | 5.77–10.35 | 2002–2005 | 41.45* | 18.41–92.07 | 2005–2021 | 2.76 | -0.32–4.76 |
| Leukemia | 5.08* | 2.85–7.27 | 2002–2005 | 37.38* | 14.98–86.36 | 2005–2021 | -0.07 | -2.97–1.85 |
| Thyroid cancer | 6.33* | 4.12–8.51 | 2002–2005 | 39.94* | 17.43–89.26 | 2005–2021 | 1.00 | -1.86–2.93 |
| Breast cancer | 6.45* | 4.17–8.72 | 2002–2005 | 38.68* | 15.63–88.65 | 2005–2021 | 1.30 | -1.76–3.25 |
| Pancreatic cancer | 10.47* | 8.01–13.51 | 2002–2005 | 47.24* | 19.66–110.67 | 2005–2021 | 4.67 | 1.47–6.81 |
| Non–Hodgkin lymphoma | 5.81* | 3.58–8.00 | 2002–2005 | 37.05* | 14.82–84.92 | 2005–2021 | 0.81 | -2.19–2.72 |
| Multiple myeloma | 6.54* | 4.22–8.87 | 2002–2005 | 37.41* | 14.48–86.90 | 2005–2021 | 1.57 | -1.73–3.51 |
| Gallbladder and biliary tract cancer | 5.72* | 3.44–7.94 | 2002–2005 | 36.48* | 14.19–84.66 | 2005–2021 | 0.78 | -2.38–2.71 |
| Ovarian cancer | 6.14* | 3.91–8.40 | 2002–2005 | 38.59* | 15.91–88.25 | 2005–2021 | 0.96 | -1.98–2.91 |
| Kidney cancer | 6.07* | 3.86–8.26 | 2002–2005 | 40.08* | 17.51–90.32 | 2005–2021 | 0.68 | -2.16–2.61 |

Note: * indicates that the APC or AAPC significantly differs from 0 at the alpha=0.05 level, and CI means the 95% confidence interval.

# **eTable 4 Temporal Trends for Different Income Tiers**

|  |  |  | Joinpoint Analysis (2002–2021) | | | | | |
| --- | --- | --- | --- | --- | --- | --- | --- | --- |
| **Income Tiers** | **AAPC** | | Trend 1 | | | Trend 2 | | |
|  | **Value** | **CI** | Year | APC | CI | Year | APC | CI |
| World Bank High Income | 6.55* | 1.79–9.52 | 2002–2004 | 119.75* | 25.03–195.57 | 2004–2021 | -2.15 | -6.94–0.74 |
| World Bank Upper Middle Income | 10.25* | 7.11–13.46 | 2002–2005 | 56.44* | 23.83–134.67 | 2005–2021 | 3.27 | -0.85–5.89 |
| World Bank Lower Middle Income | 9.69* | 7.26–11.94 | 2002–2006 | 36.27* | 19.81–73.45 | 2006–2021 | 3.52 | -0.29–6.12 |
| World Bank Low Income | 5.24* | 3.19–7.11 | 2002–2005 | 33.29* | 13.70–74.07 | 2005–2021 | 0.68 | -2.14–2.48 |

Note: * indicates that the APC or AAPC significantly differs from 0 at the alpha=0.05 level, and CI means the 95% confidence interval.

# **eTable 5 Types of Cancer with the Highest and Lowest Loss**

| **Region** | **Highest Loss** | | **Lowest Loss** | |
| --- | --- | --- | --- | --- |
|  | 2002 | 2021 | 2002 | 2021 |
| Global | Colon and rectum cancer | Colon and rectum cancer | Pancreatic cancer | Thyroid cancer |
| World Bank High Income | Colon and rectum cancer | Colon and rectum cancer | Thyroid cancer | Thyroid cancer |
| World Bank Upper Middle Income | Colon and rectum cancer | Colon and rectum cancer | Pancreatic cancer | Thyroid cancer |
| World Bank Lower Middle Income | Colon and rectum cancer | Colon and rectum cancer | Pancreatic cancer | Pancreatic cancer |
| World Bank Low Income | Liver cancer | Colon and rectum cancer | Pancreatic cancer | Pancreatic cancer |

# **eTable 6 Health and Economic Burden**

|  |  | Deaths Rate | | | | | | DALYs Rate | | | | | | Economic Loss (million $) | | | | | | |
| --- | --- | --- | --- | --- | --- | --- | --- | --- | --- | --- | --- | --- | --- | --- | --- | --- | --- | --- | --- | --- |
|  |  | 2002 | | | 2021 | | | 2002 | | | 2021 | | | 2002 | | | 2021 | | | |
| Region/Cancer Types | Sex | Value | Upper UI (95%) | Lower UI (95%) | Value | Upper UI (95%) | Lower UI (95%) | Value | Upper UI (95%) | Lower UI (95%) | Value | Upper UI (95%) | Lower UI (95%) | Value | Upper UI (95%) | Lower UI (95%) | Value | Upper UI (95%) | Lower UI (95%) |  |
| **Region** |  |  |  |  |  |  |  |  |  |  |  |  |  |  |  |  |  |  |  |  |
| Global | Both | 3·95 | 6·42 | 1·59 | 4·18 | 6·80 | 1·71 | 95·00 | 151·93 | 39·43 | 102·17 | 165·02 | 43·24 | 9301·28 | 14795·86 | 3931·56 | 3741285·47 | 44132·37 | 11510·56 |  |
| Global | Female | 4·56 | 7·52 | 1·76 | 4·67 | 7·69 | 1·83 | 110·12 | 179·32 | 43·98 | 114·61 | 185·86 | 46·08 | 5647·06 | 9158·75 | 2291·22 | 2191260·21 | 26258·13 | 6427·76 |  |
| Global | Male | 3·17 | 5·09 | 1·42 | 3·60 | 5·85 | 1·58 | 77·57 | 122·93 | 35·38 | 88·18 | 143·40 | 39·36 | 3654·21 | 5745·83 | 1684·30 | 1550025·27 | 18142·03 | 4963·05 |  |
| World Bank High Income | Both | 6·63 | 10·88 | 2·57 | 6·05 | 9·76 | 2·57 | 160·22 | 260·15 | 63·43 | 144·97 | 232·59 | 62·35 | 18129·97 | 29525·75 | 7110·17 | 44811·06 | 73389·28 | 17955·74 |  |
| World Bank High Income | Female | 7·00 | 11·77 | 2·51 | 6·63 | 11·11 | 2·47 | 168·65 | 279·78 | 61·13 | 160·53 | 262·62 | 61·63 | 10585·74 | 17659·84 | 3807·98 | 25401·01 | 42290·52 | 9516·83 |  |
| World Bank High Income | Male | 6·09 | 10·03 | 2·62 | 6·38 | 10·49 | 2·52 | 148·96 | 241·93 | 64·59 | 153·44 | 248·97 | 62·90 | 7544·23 | 12272·43 | 3255·37 | 19410·04 | 31287·30 | 8276·60 |  |
| World Bank Upper–middle Income | Both | 3·19 | 5·09 | 1·37 | 3·40 | 5·73 | 1·54 | 83·78 | 131·95 | 37·12 | 92·73 | 157·45 | 42·70 | 1588·43 | 2487·06 | 717·06 | 4615·44 | 7521·37 | 1978·33 |  |
| World Bank Upper–middle Income | Female | 3·87 | 6·29 | 1·61 | 4·44 | 7·33 | 1·80 | 100·99 | 162·66 | 43·18 | 115·94 | 191·10 | 47·92 | 985·09 | 1576·34 | 428·46 | 2703·25 | 4475·29 | 1090·80 |  |
| World Bank Upper–middle Income | Male | 2·39 | 3·72 | 1·14 | 3·96 | 6·47 | 1·70 | 64·59 | 99·76 | 31·51 | 105·19 | 170·72 | 45·96 | 603·34 | 932·44 | 297·39 | 1912·19 | 3263·95 | 872·68 |  |
| World Bank Lower–middle Income | Both | 1·40 | 2·19 | 0·62 | 2·03 | 3·31 | 0·85 | 37·47 | 57·52 | 17·28 | 53·41 | 85·97 | 23·47 | 399·83 | 621·06 | 186·69 | 2732·99 | 4291·78 | 1175·52 |  |
| World Bank Lower–middle Income | Female | 1·76 | 2·81 | 0·73 | 2·50 | 4·14 | 0·98 | 46·81 | 72·72 | 20·97 | 65·46 | 105·45 | 27·01 | 261·85 | 410·57 | 116·39 | 1793·04 | 2828·12 | 731·50 |  |
| World Bank Lower–middle Income | Male | 1·00 | 1·56 | 0·50 | 1·50 | 2·39 | 0·68 | 27·33 | 42·66 | 13·71 | 40·15 | 64·06 | 18·60 | 137·98 | 210·16 | 71·27 | 939·95 | 1460·45 | 452·87 |  |
| World Bank Low Income | Both | 1·60 | 2·56 | 0·69 | 2·18 | 3·50 | 0·89 | 42·91 | 67·84 | 19·01 | 58·16 | 92·05 | 24·49 | 45·34 | 2487·06 | 717·06 | 121·70 | 194·15 | 57·17 |  |
| World Bank Low Income | Female | 2·14 | 3·45 | 0·86 | 2·81 | 4·57 | 1·08 | 57·00 | 90·76 | 23·93 | 74·90 | 118·91 | 29·88 | 28·68 | 1576·34 | 428·46 | 75·74 | 119·32 | 33·82 |  |
| World Bank Low Income | Male | 1·03 | 1·60 | 0·52 | 1·49 | 2·34 | 0·72 | 28·49 | 43·73 | 14·34 | 40·27 | 62·72 | 19·34 | 16·66 | 932·44 | 297·39 | 45·95 | 73·26 | 21·95 |  |
| **Type of Cancer** |  |  |  |  |  |  |  |  |  |  |  |  |  |  |  |  |  |  |  |  |
| Breast cancer | Both | 0·53 | 1·07 | –0·02 | 0·52 | 1·03 | –0·02 | 11·72 | 23·50 | –0·41 | 11·63 | 22·68 | –0·49 | 1058·97 | 2115·59 | –49·66 | 3194·62 | 6226·39 | –123·38 |  |
| Breast cancer | Female | 0·95 | 1·91 | –0·03 | 0·95 | 1·89 | –0·03 | 21·85 | 43·76 | –0·81 | 21·83 | 42·51 | –1·01 | 1058·97 | 2115·59 | –49·66 | 3194·62 | 6226·39 | –123·38 |  |
| Colon and rectum cancer | Both | 1·18 | 1·89 | 0·51 | 1·17 | 1·87 | 0·51 | 26·71 | 42·50 | 11·43 | 27·33 | 43·37 | 11·80 | 2593·16 | 4119·61 | 1109·04 | 7254·52 | 11511·75 | 3134·13 |  |
| Colon and rectum cancer | Female | 1·10 | 1·77 | 0·48 | 1·04 | 1·65 | 0·45 | 24·64 | 39·52 | 10·61 | 23·96 | 37·75 | 10·36 | 1270·19 | 2035·69 | 546·43 | 3361·71 | 5301·17 | 1455·07 |  |
| Colon and rectum cancer | Male | 1·26 | 2·03 | 0·54 | 1·33 | 2·13 | 0·57 | 28·98 | 45·96 | 12·27 | 31·09 | 49·47 | 13·36 | 1322·97 | 2099·57 | 557·83 | 3892·81 | 6204·14 | 1671·45 |  |
| Gallbladder and biliary tract cancer | Both | 0·24 | 0·33 | 0·17 | 0·24 | 0·33 | 0·16 | 5·32 | 7·23 | 3·70 | 5·20 | 7·17 | 3·56 | 514·05 | 698·10 | 358·78 | 1385·12 | 1906·97 | 945·49 |  |
| Gallbladder and biliary tract cancer | Female | 0·29 | 0·39 | 0·20 | 0·26 | 0·37 | 0·18 | 6·33 | 8·61 | 4·37 | 5·84 | 8·18 | 3·94 | 325·30 | 442·59 | 224·83 | 823·54 | 1153·63 | 555·95 |  |
| Gallbladder and biliary tract cancer | Male | 0·19 | 0·26 | 0·13 | 0·21 | 0·30 | 0·14 | 4·18 | 5·73 | 2·83 | 4·51 | 6·48 | 2·94 | 188·75 | 258·10 | 126·97 | 561·57 | 805·21 | 364·13 |  |
| Kidney cancer | Both | 0·36 | 0·58 | 0·14 | 0·38 | 0·62 | 0·15 | 8·85 | 14·06 | 3·50 | 8·99 | 14·51 | 3·68 | 870·55 | 1381·88 | 344·75 | 2397·94 | 3866·50 | 979·85 |  |
| Kidney cancer | Female | 0·26 | 0·42 | 0·10 | 0·26 | 0·41 | 0·10 | 6·12 | 9·76 | 2·44 | 5·81 | 9·20 | 2·34 | 316·60 | 504·46 | 125·97 | 817·95 | 1295·32 | 329·77 |  |
| Kidney cancer | Male | 0·48 | 0·78 | 0·19 | 0·53 | 0·86 | 0·21 | 11·85 | 18·89 | 4·68 | 12·52 | 20·38 | 5·09 | 553·95 | 880·31 | 218·52 | 1580·00 | 2571·88 | 642·99 |  |
| Leukemia | Both | 0·34 | 0·44 | 0·26 | 0·32 | 0·42 | 0·23 | 9·38 | 12·15 | 7·08 | 8·72 | 11·24 | 6·56 | 973·04 | 1259·01 | 735·34 | 2273·89 | 2930·87 | 1711·63 |  |
| Leukemia | Female | 0·30 | 0·38 | 0·22 | 0·27 | 0·35 | 0·19 | 8·37 | 10·83 | 6·21 | 7·60 | 9·88 | 5·51 | 451·81 | 583·72 | 334·54 | 1028·99 | 1336·34 | 744·64 |  |
| Leukemia | Male | 0·40 | 0·52 | 0·30 | 0·38 | 0·50 | 0·28 | 10·58 | 13·82 | 7·94 | 10·02 | 13·14 | 7·41 | 521·23 | 684·23 | 392·23 | 1244·89 | 1632·71 | 921·17 |  |
| Liver cancer | Both | 0·37 | 0·59 | 0·15 | 0·53 | 0·90 | 0·21 | 9·87 | 15·83 | 4·10 | 14·16 | 24·06 | 5·77 | 1000·03 | 1596·28 | 415·94 | 3795·93 | 6448·57 | 1546·95 |  |
| Liver cancer | Female | 0·27 | 0·45 | 0·11 | 0·38 | 0·64 | 0·15 | 6·77 | 11·18 | 2·71 | 9·52 | 15·91 | 3·91 | 353·10 | 582·96 | 141·33 | 1330·91 | 2228·17 | 546·94 |  |
| Liver cancer | Male | 0·47 | 0·76 | 0·20 | 0·70 | 1·21 | 0·29 | 13·08 | 21·00 | 5·55 | 19·05 | 33·00 | 7·83 | 646·93 | 1037·17 | 274·43 | 2465·02 | 4269·09 | 1013·85 |  |
| Multiple myeloma | Both | 0·10 | 0·25 | –0·04 | 0·11 | 0·27 | –0·04 | 2·18 | 5·53 | –0·79 | 2·39 | 5·94 | –0·97 | 209·94 | 531·46 | –76·72 | 637·00 | 1581·42 | –258·74 |  |
| Multiple myeloma | Female | 0·09 | 0·23 | –0·03 | 0·10 | 0·24 | –0·04 | 2·03 | 5·00 | –0·77 | 2·17 | 5·37 | –0·91 | 104·22 | 256·47 | –39·67 | 306·14 | 758·33 | –129·38 |  |
| Multiple myeloma | Male | 0·11 | 0·27 | –0·04 | 0·12 | 0·32 | –0·05 | 2·35 | 5·97 | –0·84 | 2·66 | 6·89 | –1·06 | 105·72 | 267·90 | –37·86 | 330·86 | 856·00 | –132·96 |  |
| Non–Hodgkin lymphoma | Both | 0·15 | 0·26 | 0·05 | 0·15 | 0·26 | 0·05 | 3·92 | 6·68 | 1·30 | 4·00 | 6·90 | 1·33 | 394·48 | 670·84 | 131·26 | 1054·06 | 1820·52 | 351·50 |  |
| Non–Hodgkin lymphoma | Female | 0·13 | 0·23 | 0·04 | 0·13 | 0·23 | 0·04 | 3·32 | 5·70 | 1·11 | 3·38 | 5·83 | 1·12 | 174·38 | 299·36 | 58·14 | 466·79 | 804·75 | 154·31 |  |
| Non–Hodgkin lymphoma | Male | 0·17 | 0·29 | 0·06 | 0·18 | 0·31 | 0·06 | 4·56 | 7·71 | 1·52 | 4·67 | 8·01 | 1·53 | 220·10 | 371·20 | 73·11 | 587·27 | 1005·53 | 192·01 |  |
| Ovarian cancer | Both | 0·20 | 0·36 | 0·04 | 0·20 | 0·36 | 0·05 | 5·09 | 9·29 | 1·10 | 5·46 | 9·62 | 1·30 | 508·48 | 929·62 | 108·98 | 1464·14 | 2577·03 | 348·05 |  |
| Ovarian cancer | Female | 0·36 | 0·66 | 0·08 | 0·38 | 0·67 | 0·09 | 9·77 | 17·84 | 2·10 | 10·56 | 18·57 | 2·50 | 508·48 | 929·62 | 108·98 | 1464·14 | 2577·03 | 348·05 |  |
| Pancreatic cancer | Both | 0·05 | 0·20 | –0·04 | 0·11 | 0·31 | –0·03 | 1·22 | 4·71 | –0·95 | 2·54 | 7·16 | –0·56 | 118·20 | 457·53 | –92·50 | 685·27 | 1922·18 | –146·59 |  |
| Pancreatic cancer | Female | 0·07 | 0·22 | –0·03 | 0·12 | 0·31 | –0·01 | 1·55 | 4·75 | –0·59 | 2·75 | 7·11 | –0·24 | 79·79 | 244·35 | –30·48 | 389·48 | 1005·35 | –33·47 |  |
| Pancreatic cancer | Male | 0·03 | 0·19 | –0·06 | 0·09 | 0·30 | –0·04 | 0·80 | 4·63 | –1·46 | 2·28 | 7·24 | –0·92 | 38·40 | 217·38 | –67·04 | 295·79 | 930·19 | –114·34 |  |
| Thyroid cancer | Both | 0·06 | 0·07 | 0·04 | 0·06 | 0·08 | 0·05 | 1·55 | 1·97 | 1·18 | 1·68 | 2·14 | 1·26 | 155·84 | 197·43 | 118·28 | 444·71 | 566·78 | 335·10 |  |
| Thyroid cancer | Female | 0·07 | 0·09 | 0·05 | 0·07 | 0·09 | 0·05 | 1·89 | 2·43 | 1·43 | 1·96 | 2·55 | 1·44 | 99·68 | 128·01 | 75·38 | 270·34 | 351·18 | 199·39 |  |
| Thyroid cancer | Male | 0·04 | 0·06 | 0·03 | 0·05 | 0·07 | 0·04 | 1·18 | 1·50 | 0·88 | 1·38 | 1·79 | 1·04 | 56·16 | 71·39 | 42·39 | 174·36 | 225·30 | 130·94 |  |
| Uterine cancer | Both | 0·36 | 0·48 | 0·26 | 0·39 | 0·50 | 0·28 | 9·20 | 12·11 | 6·52 | 10·07 | 13·28 | 7·22 | 904·54 | 1191·11 | 640·00 | 2700·19 | 3561·60 | 1936·34 |  |
| Uterine cancer | Female | 0·66 | 0·88 | 0·47 | 0·72 | 0·94 | 0·52 | 17·47 | 22·99 | 12·37 | 19·23 | 25·38 | 13·80 | 904·54 | 1191·11 | 640·00 | 2700·19 | 3561·60 | 1936·34 |  |

**Note:** The death and DALY rates here are age-standard indexes that control the effect of population size and age composition. Economic loss is calculated from the total DALYs instead of age-standardized DALYs shown in the table, considering the influence of population growth and age structure.
